# Skin injury model classification based on shape vector analysis

**DOI:** 10.1186/1471-2342-12-32

**Published:** 2012-11-06

**Authors:** Emil Röhrich, Michael Thali, Wolf Schweitzer

**Affiliations:** 1 Institute of Forensic Medicine, University of Zürich, Winterthurerstr. 190/52, 8057 Zürich, Switzerland

## Abstract

**Background:** Skin injuries can be crucial in judicial decision making. Forensic experts base their classification on subjective opinions. This study investigates whether known classes of simulated skin injuries are correctly classified statistically based on 3D surface models and derived numerical shape descriptors.

**Methods:** Skin injury surface characteristics are simulated with plasticine. Six injury classes – abrasions, incised wounds, gunshot entry wounds, smooth and textured strangulation marks as well as patterned injuries - with 18 instances each are used for a *k*-fold cross validation with six partitions. Deformed plasticine models are captured with a 3D surface scanner. Mean curvature is estimated for each polygon surface vertex. Subsequently, distance distributions and derived aspect ratios, convex hulls, concentric spheres, hyperbolic points and Fourier transforms are used to generate 1284-dimensional shape vectors. Subsequent descriptor reduction maximizing SNR (signal-to-noise ratio) result in an average of 41 descriptors (varying across *k*-folds). With non-normal multivariate distribution of heteroskedastic data, requirements for LDA (linear discriminant analysis) are not met. Thus, shrinkage parameters of RDA (regularized discriminant analysis) are optimized yielding a best performance with *λ* = 0.99 and *γ* = 0.001.

**Results:** Receiver Operating Characteristic of a descriptive RDA yields an ideal Area Under the Curve of 1*.*0for all six categories. Predictive RDA results in an average CRR (correct recognition rate) of 97,22% under a 6 partition *k*-fold. Adding uniform noise within the range of one standard deviation degrades the average CRR to 71,3%.

**Conclusions:** Digitized 3D surface shape data can be used to automatically classify idealized shape models of simulated skin injuries. Deriving some well established descriptors such as histograms, saddle shape of hyperbolic points or convex hulls with subsequent reduction of dimensionality while maximizing SNR seem to work well for the data at hand, as predictive RDA results in CRR of 97,22%. Objective basis for discrimination of non-overlapping hypotheses or categories are a major issue in medicolegal skin injury analysis and that is where this method appears to be strong. Technical surface quality is important in that adding noise clearly degrades CRR.

**Trial registration:** This study does not cover the results of a controlled health care intervention as only plasticine was used. Thus, there was no trial registration.

## Background

**Figure 1:**
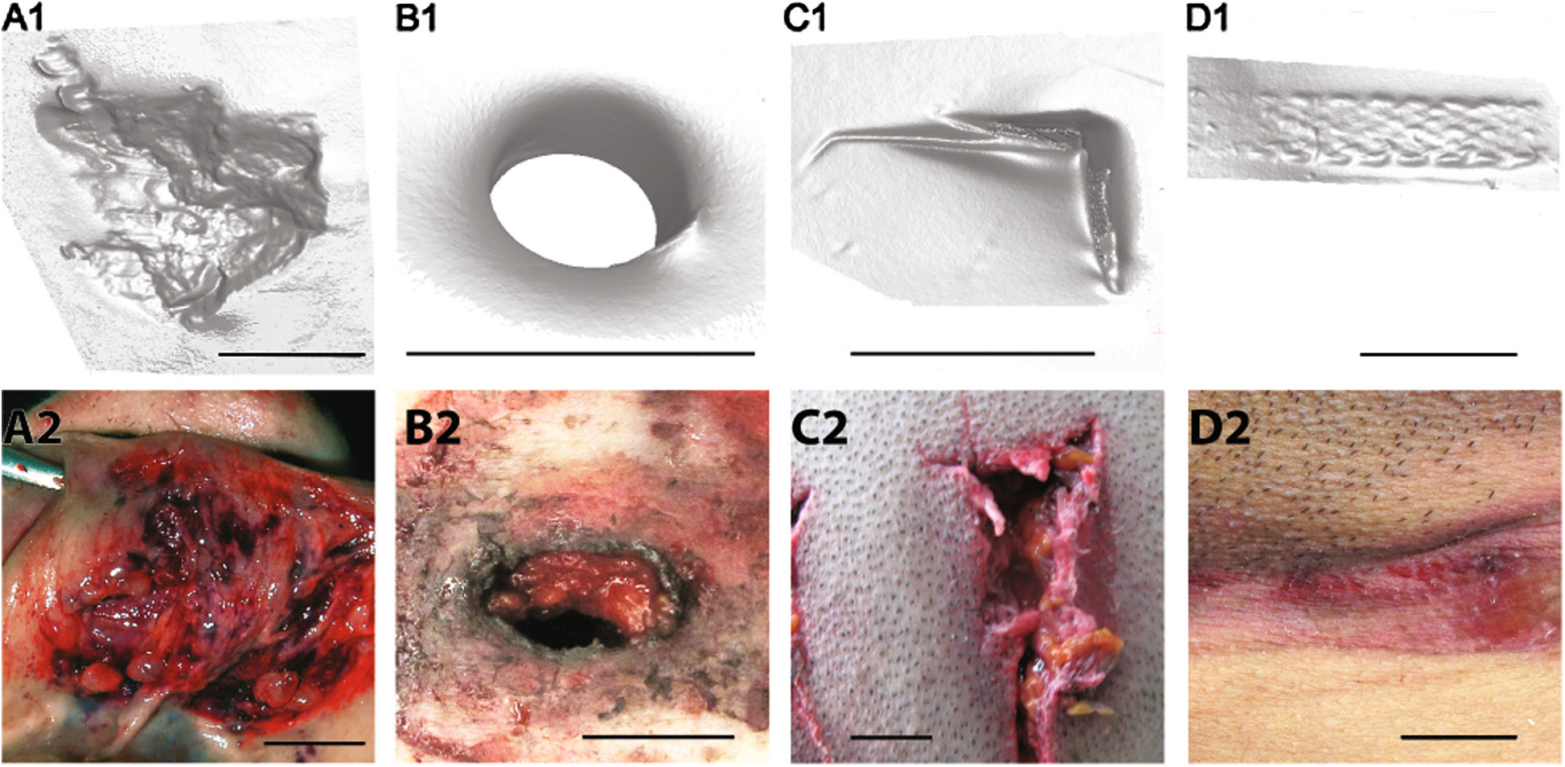
**Plasticine models versus real injuries.** Surface geometry of injured skin surface (bottom image row) was modelled using plasticine blocks that were then digitized (top image row). Abrasions during sliding or impacting on rough surface such as roads or walls typically result in an irregularly shaped injury surface **(A2)** that can contain groves as well as rounded indented or protruding features, often with apparently poor delineation. These are simulated by applying similar rough flat surface structures to plasticine **(A1)**. Such injuries typically occur in road traffic accidents, building or reconstruction sites or falls. Gunshot entry wounds **(B2)** are geometrically simulated with circular penetrating defects to a plasticine block **(B1)**. Patterned lacerations **(C2)** typically combine delineated edgy boundaries and indented or protruding skin flaps where straight edges might still be identifiable. Using a longitudinal sharp-edged object, similar wound features result on plasticine **(C1)**. Such injuries are found after using sharply edged objects such as for example metal covers found in buildings or ventilation funnels, on trains or other vehicles. Strangulation marks **(D2)** contain a longitudinal groove whose ’valley’ surface may or may not exhibit a finely striated substructure. This can reflect indentation of the skin by a rope-like structure such as textured strangulation marks simulated in our plasticine model **(D1)**. Bar length is 1 cm in all images.

A core task in forensic medicine is the classification of skin injuries such as illustrated in the bottom row of Figure 1. Injuries are classified to estimate their possible sequence over time, possible causes, possible age or other details of the events that may have accompanied their genesis. Absence of an injury’s membership to a particular diagnostic group can be relevant. Also, a ’specificity paradox’ [1] must be avoided – just because a particular injury can be examined from very close up and high quality photo prints are available its cause does not necessarily become established in a more precise way.

Forensic experts’ opinions regarding classification of injuries have to be considered state of the art but may differ according to individually variable experience or skill, they may be unstable over time or even inadequately stable (injury re-interpretation may have to achieve different results if new information becomes available, for example). Experts’ opinions appear to depend on external conditions such as lighting [2], or they may be influenced by strategic considerations. Furthermore, admitting that a particular weapon-injury comparison is not conclusive at all may be hard for some experts. All of that still has to be accepted as essential part of forensic medicine: interpretation of injuries can be conflicting, particularly where an objective basis for finding conclusions is absent. It is not the duty of experts to necessarily agree in any such an opinion but there can be significant suffering and cost as result. These concerns are neither new nor solved [3].

What is clear is that it is the duty of forensic and medico-legal experts alike to provide an objective basis for later deliberations wherever possible. With a properly set up automated system, an objective basis for wound categorization can be explained, documented, shown, modified, and new additional information may alter, confirm or leave open any present or absent membership of a questioned injury to a particular diagnostic group. Such a tool may be of useful assistance to the practice of forensic medicine. Capturing by 2D or 3D optical techniques and manual reconstructive juxtapositioning of relevant two-dimensional projection or cross section of weapon and skin injury – assuming a non-deformed skin imprint of the injury causing tool or weapon – for the medico-legal illustration of match plausibility was first published by Werkgartner (1935) [4] and later also applied to 3D documentation using photogrammetric or other 3D capturing methods [5-10]. However, matching elastically deformed tool or weapon imprints on basis of shape encoding and subsequent statistical methods has not been established for medico-legal investigation of injuries so far.

Objective classification methods using machine based shape recognition nowadays can yield good results in a number of fields [11], including the discrimination of biologic shapes and the discrimination of shapes typically compared in forensic sciences. In a study published 1991, automated segmentation based on curvature yielded four facial feature sets; depending on which set was used a recognition rate of 70.8% to 100.0% resulted [12]. A more recent technique for face recognition was designed to be more robust with respect to factors such as aging or illumination; the authors used weighted local binary patterns and achieved a best correct recognition rate of 97% [13]. Details of technical coding and comparison play a major role in the success of classification methods in that rigid representations including eigenvalues or eigenspectra [14] were found to be outperformed by histogram based techniques with a best classification success rate of 98.75 to 100% [15]. In one study, tool marks produced with a range of screwdrivers and pliers at different angles on various relatively hard media (lead, brass, steel, aluminum) could be automatically recognized with error rates between 0*.*00*%* and 49*.*5*%*[16]. The fractal nature of biologic shapes can be exploited for injured regions and their bounding transition areas to surrounding unharmed skin: they contain relevant geometry on multiple scale levels. In [17] dermatologists show an automatic method for discrimination between melanoma and less dangerous melanocytic nevi using two shape descriptors: lacunarity, a measure for distribution and size of holes, and fractal dimension, an irregularity measure of the shape obtained by transforming the color hue of 2D wound images into a third Cartesian coordinate. – In another promising approach, shape outlines or silhouette contours were analyzed by deriving skeleton-like information by submitting the contours to the Poisson equation; error rates were reported to range from 1*.*8 to 2*.*1*%* on 2D data [18]; the method was later also applied to medical 3D data [19]. Even at molecular levels, accurate identification of protein binding sites is reported to succed in 85% using shape descriptors [20]. Forensic evidence such as cartridge case shear marks yielded a bootstrapped 0% error rate in a descriptive study performing PCA on unpartitioned data [21].

However, practice of forensic medicine does not incorporate these techniques for routine work. Generally, the absence of widely available high-resolution digital 3D skin surface data and ill-defined specifics of shape encoding for that particular application domain appear to present a major obstacle in using shape analysis for forensic medicine. Histogram or distribution frequency based 3D descriptors have attracted considerable attention [22] so we combined a number of appealing concepts including multi scale encoding, spherical frequencies, histogram based techniques, autocorrelation and Fourier transforms in this study.

Skin injuries exhibit shape and surface characteristics that ideally can be recognized as specific for a particular injury cause. Our study uses plasticine block based representative simulations of skin injury surfaces that represent ideal examples for injury caused by a range of agents (Figure 1; explained in more detail below). We use these to answer two questions: Can shape descriptors that are automatically derived from 3D surface data be used to reliably discriminate classes of simulated skin injuries defined by respective common injury cause? What is the performance of such shape descriptors in an idealized setting?

## Methods

### Skin injury simulation using prototypes

#### Typical versus atypical injuries

Forensic pathology is a field that routinely employs pragmatic injury categories such as blunt force, sharp force, firearm injury or patterned injuries, to name a few. From that we then assume a clear distinction in the appearance of *typical* injuries whose distinct appearance, ideally, leads to their cause. In this study we model skin injuries using plasticine. We propose that these are sufficiently representative of typical, so-called textbook or ideally shaped injuries; four comparisons of typical real skin injuries and our plasticine correlates are shown in Figure 1. We resort to plasticine because that material preserves shape. Persistent surface qualities are required to arrive at a useful training set of injury shape based 3D mesh objects. Conversely, real skin injuries are not useful to start method development: *in vivo*, real skin injuries will heal, whereas *post mortem*, injury shape tends to deteriorate, dry out and decay. In both instances, characteristic features are lost over hours and days following injury [23-25]. And we need considerable time: parameters of the 3D scanner and statistical evaluation all require repeated evaluation at first. That is why we use plasticine blocks to iteratively improve 3D scanner setup, 3D mesh object consistency and optimal data quality.

It is a fact that tools or instruments can cause injury shapes that might appear to be hard or impossible to evaluate as to their true cause. Depending on their true cause they might be termed *atypical* injuries, or the tools or items used to produce them exhibit strong similarities themselves. As an example, one might argue that a superficial abrasion typically is caused by blunt force trauma. Yet, tangential sharp force such as a knife or razor blade might cause a similar appearance and also result in an abrasions whereas one could contend that sharp force usually causes incised wounds. There are injuries that can present a phenotype overlap and appear to allow no further distinction, which is an issue that is often further complicated by post mortem decay. So ultimately there might be injuries that are best classified by placing them *in between* two or three known categories.

To build classification systems also for shapes that are hard to classify, one has to start with distinct and clear, unambiguous samples that exhibit striking features. Having said that, there do exist injury shapes that have to be regarded as typical text book examples for the agent that caused them. There are injuries that are representative for a particular tool. In order to build a shape encoding method for further statistical analysis, we employed plasticine models that we judged as representative for the 3D skin surface structure of typical text book type injury categories.

#### Injury classes

For each plasticine injury model we start with a flat, straightened block of standard issue plasticine. Typical injuries are then simulated by manually pressing or hitting various tools into the flat plasticine surface. Simulated skin injuries cover the following 6 injury classes that we consider as ’typical’ for the causing instrument: 

1. *Incised wounds:* A knife and the tip of a scissors’ blade are used to produce this type of plasticine damage. – Generally, sharp force injury is defined to be caused by relatively sharply edged objects; *incised wounds* or *cuts* are differentiated from *stab wounds* in that *cuts* are by definition longer than deep while a *stab wound* is defined to be deeper than wide [23,26]. Sharp force injuries typically exhibit relatively straight wound edges, tissue bridging as seen in lacerations are absent.

2. *Abrasions:* Tearing the plasticine block across the rough surface of a paved road is employed to model abrasions. – By definition one would regard an abrasion as a tangential graze or gravel rash which may penetrate the full skin involving scattered or confluent shapes of punctuate or streaky lacerations. A typical abrasion might not be lined by straight edges.

3. *Gunshot entry wounds:* We use a standard issue pencil to pierce the plasticine block and obtain a defect that resembles a penetrating round firearm entry wound. – Gunshot entry wounds are bullet entry wounds and typically, they constitute penetrating injuries that may have the appearance of gaping holes similar to impalement injuries.

4. *Smooth strangulation marks:* We pull a power cable against the plasticine blocks to simulate smooth (non-patterned) strangulation marks. – Strangulation marks are longitudinal neck injuries on the neck caused by compression through a cord, rope, cable, wire, belt or other longitudinal flexible object. They typically contain a combination of skin indentation along with abrasion or bruising, skin drying or local mummification particularly of ridge structures that may also reflect any surface structure of the strangulation tool through exhibiting a patterned indented skin surface.

5. *Textured strangulation marks:* We apply a textile shoe lace against plasticine blocks to simulate textured strangulation marks.

6. *Patterned injuries:* We use a thin, L-shaped metal structure to generate slashes or lacerations. In forensic pathology, the term patterned injuries typically is used to denote injury shapes that are contained by defined contours. They may feature abrasions or skin indentations that feature an apparent geometrical match of a prominent part of the causing instrument’s shape. Typical patterned injuries are polygonal abrasions from a vehicle’s tyre, or complex abrasions caused by tearing a chain-like necklace to cause distinct chain patterned abrasions.

We create 108 plasticine models to obtain 6 classes of equal size. Hence, each of the injury classes described above will be represented by a group of 18 injuries. That allows us to take out three injury instances of each group to build a test set, use the remaining instances as training set, and arrive at a *k*-fold cross-validation procedure with 6 partitions.

### Digitization of plasticine models

We use a 3D surface scanner that employs collimated light patterns to capture the shape of the modeled injuries.

Adaptively refined meshes are commonly obtained as result of 3D scans. They combine surface detail for relatively uneven data with data size reduction for relatively even surface regions [27]. For this study however, spatial frequency of various vertex derived parameters are calculated for feature specification. Instead of adaptive mesh refinement, we obtain mesh objects that are evenly gridded. Resulting mesh resolution is around 10 vertices per millimetre in all directions, triangles are nearly equilateral.

Digitization of 108 plasticine blocks contains a sequence of standardized work steps (capturing 3D mesh parts, segmenting, registering, optimizing, mesh generation, mesh optimization, noise reduction). This work flow produces 3D mesh surfaces that digitally represent the plasticine models that were scanned.

**Figure 2:**
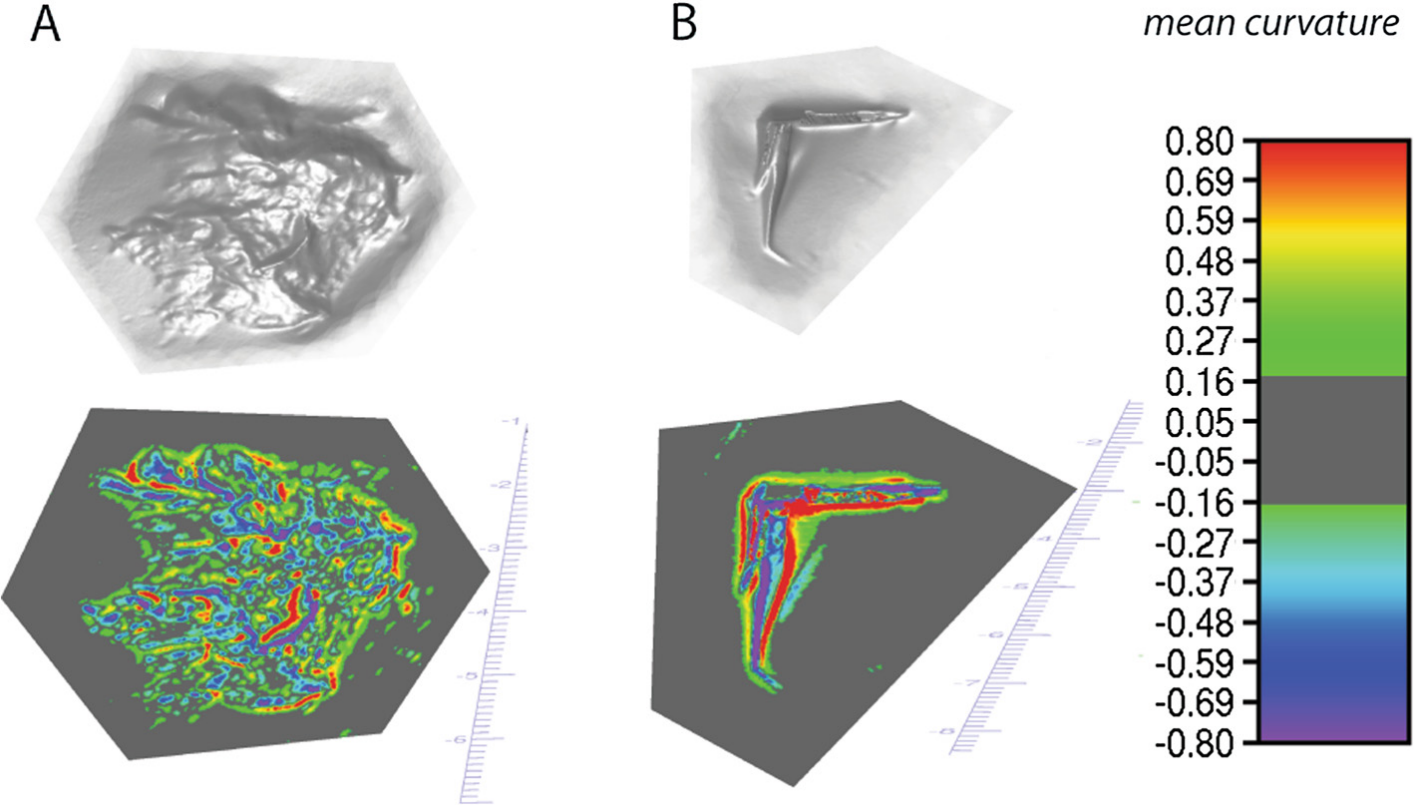
**Mesh objects and curvature maps.** Untextured 3D mesh object surfaces of plasticine models containing an abrasion (**A** top) and a patterned laceration (**B** top). At each vertex, mean curvature is mapped to a color (A bottom, B bottom; mean curvature values see color legend). Convex regions range from green (flat) to red (strongly convex) and concave regions from green to violet (strongly concave). Flat regions exhibiting a curvature magnitude below 0*.*17are dark gray and excluded from further evaluation. Ridges, grooves and shape configurations can be visually checked to be distinct for both 3D mesh objects now.

Each mesh surface is given as a data structure consisting of a 3×*n* vector of vertices and a 3×*m*vector of triangles. In each instance there is a centrally located surface feature, injury or lesion that is surrounded by peripheral flat surface. For further analysis, surrounding flat surface regions are manually clipped. Precise location and extent of the manual clipping procedure remains without impact on the results, as only convex and concave data pertaining to the central feature lesion undergo further analysis (see gray regions in Figure 2). Prior to computing all elements of the initial shape descriptor vector (Table 1), mesh clips are resized to fit into a unit square in an attempt to aim for a size independent classification of the injuries.

**Table 1:** Shape descriptors for each of the 12 curvature subsets

**Descriptors *d***_***i***_	**Description**
{*d*_0_,…,*d*_39_}	Frequencies of Euclidean distance to other vertices contained in the subset *S*_*n*_with *n*∈{0,…,11} , counted within boundaries of 40 bins.
{*d*_40_}	Ratio of maximum and mean distance between vertices [28,29].
{*d*_41_}	Ratio of maximum and median distance between vertices [28,29].
{*d*_42_}	Volume of the convex hull for the vertices set belonging to each curvature domain [30].
{*d*_43_}	Surface of the convex hull for the vertices set belonging to each curvature domain [30].
{*d*_44_}	Ratio of maximum frequency to mean frequency [28,29].
{*d*_45_}	Total number of vertices [30].
{*d*_46_,…,*d*_55_}	Number of vertices within each sphere of a series of differently sized 10 concentric spheres around the origin [31,32].
{*d*_56_,…,*d*_95_}	Fourier transform of the frequency distribution in the curvature histogram [28].
{*d*_96_,…,*d*_105_}	Fourier transform of the point distribution in the spheres series [28,31,32].
{*d*_106_}	Number of hyperbolic points [33].

Shape descriptors that are determined for each of the 12 curvature subsets (see Table 2 for curvature subset details).

### Shape descriptors

#### Descriptor definition

Curvature – as opposed to other registration point derived measures [34] such as depth maps or needle maps [35] – is a viewpoint and rotation invariant local surface characteristic. We compute curvature by estimating the per-face Weingarten matrix for each vertex [36]. A positive mean curvature value indicates local convexity, whereas locally concave regions exhibit negative mean curvature values. Resulting mean curvature can be mapped onto the 3D mesh object surface as color map; this is illustrated in Figure 2 for an abrasion (left) and a patterned laceration (right). We partition vertices of each mesh object into 13 subsets according to their approximated mean curvature values (Table 2) to achieve a type of multiscale decomposition [31]. One of these subsets *S*_*f*_is comprised of flat areas mainly found surrounding the actual injury or defect. We discard *S*_*f*_ and the remaining 12 subsets of vertices *S*_*n*_with *n*∈{0,…,11}are used for further analysis. The precise subset definitions are detailed in Table 2. Visual inspection of the mesh objects textured according to vertex curvature yielded *mc*=|0*.*17| as optimal value out of a range of |*mc*|∈[0,0*.*3]. Smaller threshold values appear to push too many vertices into subsets *S*_5_ and *S*_11_(see Table 2), thereby increasing noise levels in these subsets. Larger threshold values were found to mask regions belonging to what we regard as ’specific injury surface’; this seemed to be relevant particularly for 3D mesh objects showing low curvature magnitude. In Figure 2 the areas filtered for *S*_*f*_ are displayed gray as opposed to the colored regions exhibiting higher curvature magnitude that then are used for further analysis.

**Table 2:** Curvature subsets

**Subset**	**Description**	**Mean curvature (*mc*)**
*S*_*f*_	Flat	0*.*17<*mc*≤0*.*17
*S*_0_	Strongly convex	*mc*>0*.*8
*S*_1_	Highly convex	0*.*6<*mc*≤0*.*8
*S*_2_	Very convex	0*.*5<*mc*≤0*.*6
*S*_3_	Convex	0*.*4<*mc*≤0*.*5
*S*_4_	Slightly convex	0*.*25<*mc*≤0*.*4
*S*_5_	Little convex	0*.*17<*mc*≤0*.*25
*S*_6_	Strongly concave	*mc*<−0*.*8
*S*_7_	Highly concave	−0*.*8≤*mc*<−0*.*6
*S*_8_	Very concave	−0*.*6≤*mc*<−0*.*5
*S*_9_	Concave	−0*.*5≤*mc*<−0*.*4
*S*_10_	Slightly concave	−0*.*4≤*mc*<−0*.*25
*S*_11_	Little concave	−0*.*25≤*mc*<−0*.*17

Range of curvature values used for partitioning vertices of our 3D mesh objects into 12 subsets. These are divided in two groups: the first group, *S*_0_,…,*S*_5_, contains sets of convex spots of similar magnitude decreasing along the indices, whereas *S*_6_,…,*S*_11_analogously encompass regions of increasingly concave nature. Vertices pertaining to subset *S*_*f*_of relatively flat regions containing a mean curvature magnitude of |*mc*|≤0*.*17 are excluded from further analysis.

For each vertex subset *S*_*n*_with *n*∈{0,…,11}, a range of descriptors are derived from vertex properties. A shape vector is assembled using a range *i*∈[1,106] of descriptors *d*_*i*_. We start injury descriptor generation by determining all inter-vertex distances for every subset. Distance frequencies are split into 40 equally sized bins and stored as the first 40 vector elements of the shape vector. We then derive a range of further descriptors based on aspect ratios [28-30], convex hulls [30], concentric spheres [31,32], hyperbolic points [33] and Fourier transforms [28]. Altogether we obtain 107 shape descriptors. Technical details and references for our shape descriptors are listed in Table 1. As we do that for every of the twelve curvature domains (see Table 2), the final shape vector for each injury model amounts to a total of 12×107=1284 shape vector elements.

This process yields a relatively large initial shape vector that then is narrowed down by selecting elements with particularly promising discriminatory characteristics.

#### Descriptor reduction

Descriptor count reduction is based on analysis of the training sets’ vector elements (see next section for details regarding the k-folds).

The goal of reduction of dimensionality is a better discrimination of subsequent DA (discriminant analysis) [37], stability and performance. Cross correlation of vector elements is an impediment to DA. Furthermore, LDA (linear DA) assumes multivariate normality. To narrow down descriptor elements to the ones with the best discriminatory power, we thus remove descriptor elements that fall short of threshold values for an SNR (signal-to-noise ratio) value, and that exceed threshold values for skewness, for kurtosis and for the Kendall’s *τ*_*b*_cross correlation coefficient. This follows a Feature Selection strategy of filtering [38]. 

• With 6 injury groups there are 15 pairs of groups. For each pair of groups an SNR for each vector element variable is computed. The difference of the means of each variable over each group to another group is divided by the maximum scatter of the analyzed variable across both groups. We discard descriptors that show an SNR of lower than an arbitrarily selected threshold of 3*.*00.

• For all 6 injury groups, kurtosis and skewness are obtained for each vector element. The highest of these six results is evaluated for each vector element: vector elements whose kurtosis exceed a threshold of 16*.*0or whose skewness exceed a threshold of 13*.*4are discarded.

• Kendall’s *τ*_*b*_correlation coefficient is used to exclude excessive cross correlation for all variable combinations exhibiting *τ*_*b*_>0*.*95.

Varying across k-folds, descriptior subsets with an average of 41*.*5±4*.*9 (35 to 49) descriptors remains. More details are given in Table 3.

**Table 3:** ***k***-fold derived shape vector elements

***i***	***SV***_***i***_***Par***_**1**_	***SV***_***i***_***Pa r***_**2**_	***SV***_***i***_***Pa r***_**3**_	***SV***_***i***_***Pa r***_**4**_	***SV***_***i***_***Pa r***_**5**_	***SV***_***i***_***Pa r***_**6**_
1	*c*_5_*ca d*_96_	*c*_5_*ca d*_96_	*c*_5_*ca d*_96_	*c*_5_*ca d*_96_	*c*_5_*ca d*_96_	***c***_**5**_***ca d***_**106**_
2	*c*_4_*ca d*_94_	*c*_4_*ca d*_106_	*c*_5_*ca d*_52_	*c*_5_*ca d*_51_	*c*_4_*ca d*_106_	*c*_5_*ca d*_96_
3	*c*_4_*ca d*_58_	*c*_4_*ca d*_96_	*c*_5_*ca d*_43_	*c*_4_*ca d*_96_	*c*_4_*ca d*_96_	*c*_4_*ca d*_51_
4	*c*_4_*ca d*_43_	*c*_4_*ca d*_52_	*c*_4_*ca d*_42_	*c*_4_*ca d*_52_	*c*_4_*ca d*_59_	*c*_4_*ca d*_49_
5	*c*_3_*ca d*_94_	*c*_3_*ca d*_42_	*c*_4_*ca d*_43_	*c*_4_*ca d*_43_	*c*_4_*ca d*_52_	*c*_4_*ca d*_48_
6	*c*_3_*ca d*_43_	*c*_2_*ca d*_43_	*c*_3_*ca d*_96_	*c*_3_*ca d*_106_	*c*_4_*ca d*_43_	*c*_3_*ca d*_106_
7	*c*_1_*ca d*_52_	*c*_1_*ca d*_43_	*c*_3_*ca d*_43_	*c*_2_*ca d*_43_	*c*_4_*ca d*_42_	*c*_2_*ca d*_106_
8	*c*_1_*ca d*_45_	*c*_0_*ca d*_43_	*c*_2_*ca d*_106_	*c*_1_*ca d*_43_	*c*_2_*ca d*_43_	*c*_2_*ca d*_43_
9	*c*_1_*ca d*_43_	*c*_0_*ca d*_41_	*c*_2_*ca d*_43_	*c*_0_*ca d*_41_	*c*_2_*ca d*_42_	*c*_1_*ca d*_43_
10	*c*_0_*ca d*_51_	*c*_5_*cx d*_90_	*c*_2_*ca d*_42_	*c*_0_*ca d*_40_	*c*_1_*ca d*_53_	*c*_0_*ca d*_43_
11	*c*_0_*ca d*_43_	*c*_5_*cx d*_53_	*c*_1_*ca d*_106_	*c*_5_*cx d*_106_	*c*_0_*ca d*_41_	*c*_0_*ca d*_41_
12	*c*_0_*ca d*_41_	*c*_5_*cx d*_52_	*c*_1_*ca d*_43_	*c*_5_*cx d*_96_	*c*_0_*ca d*_40_	*c*_0_*ca d*_40_
13	*c*_0_*ca d*_40_	*c*_5_*cx d*_48_	*c*_0_*ca d*_106_	*c*_5_*cx d*_90_	*c*_5_*cx d*_105_	*c*_5_*cx d*_106_
14	*c*_5_*cx d*_106_	*c*_5_*cx d*_45_	*c*_0_*ca d*_43_	*c*_5_*cx d*_53_	*c*_5_*cx d*_90_	*c*_5_*cx d*_96_
15	*c*_5_*cx d*_64_	*c*_5_*cx d*_43_	*c*_0_*ca d*_41_	*c*_5_*cx d*_52_	*c*_5_*cx d*_52_	*c*_5_*cx d*_88_
16	*c*_5_*cx d*_63_	*c*_5_*cx d*_42_	*c*_0_*ca d*_40_	*c*_5_*cx d*_48_	*c*_5_*cx d*_48_	*c*_5_*cx d*_63_
17	*c*_5_*cx d*_62_	*c*_5_*cx d*_41_	*c*_5_*cx d*_106_	*c*_5_*cx d*_43_	*c*_5_*cx d*_45_	*c*_5_*cx d*_62_
18	*c*_5_*cx d*_53_	*c*_4_*cx d*_106_	*c*_5_*cx d*_96_	*c*_5_*cx d*_42_	*c*_5_*cx d*_43_	*c*_5_*cx d*_53_
19	*c*_5_*cx d*_52_	*c*_4_*cx d*_96_	*c*_5_*cx d*_90_	*c*_5_*cx d*_41_	*c*_5_*cx d*_42_	*c*_5_*cx d*_48_
20	*c*_5_*cx d*_50_	*c*_4_*cx d*_52_	*c*_5_*cx d*_49_	*c*_5_*cx d*_40_	*c*_4_*cx d*_106_	*c*_5_*cx d*_43_
Total	49	35	42	39	39	45

Resulting top 20 shape vector elements are listed for each of the six *k*-folds (six columns), total shape vector element dimension is shown at the bottom of each column; that figure is based on a range of thresholds (SNR, kurtosis, skewness, Kendall’s *τ *). The abbreviated naming of vector elements represents a specific combination of coarseness of curvature scale (*c*_*0*_*,…,c*_*5*_), curvature type (convex *cx *, concave *ca *) and an index referring to a derived shape descriptor described in Table1.

#### ***K***-fold cross validation

Under the *k*-fold cross validation concept, the whole database of 108 simulated skin injuries is split into a training set and a test set. We use 6 such *k*-fold partitions. Training set consists of 90 skin injuries and the test set of the remaining 18 skin injuries. For each *k*-fold partition, the test set is changed. This effectively avoids the technical errors contained in single-step or step-wise selection of classifiers based on the whole set of observations before splitting it into training and test set [39].

### Discriminant analysis

Discriminant analysis (DA) is a technique commonly used in classification problems. Given a set of groups containing known members one wants to establish how likely a particular group membership for a new and not yet classified object is.

#### Data pre-conditioning

In order to eliminate effects of scale we standardize the variables to have their mean value equal to 0 and their standard deviation equal to 1.

#### Outliers

Initially, we identify two outliers that are found to exhibit technically deficient mesh data: one is insufficiently clipped, the other appears to contain surface spikes possibly as a result of reflection artifacts at 3D scanning. After repairing these meshes, no outliers remained.

#### Multivariate normal distribution

Using Shapiro-Wilks test for our data, we establish a significant result for absence of multivariate normal distribution (group specific p-values far below 0.01). Data violating the normality assumption not due to outliers may still be suitable for particular techniques of DA such as RDA but a higher misclassification rate can result [40].

#### Homoskedasticity assumption

LDA (linear discriminant analysis) assumes homoskedasticity [41], ([42], e.g. p. 279). Several variables show different standard deviations of SNR between groups, which points to differences in covariance matrices. This difference is uneven between variables, so we cannot assume a proportional relationship. For the case of unequal covariance matrices the RDA (regularized discriminant analysis) or QDA (quadratic discriminant analysis) is recommended.

#### LDA, RDA and QDA

Our data does neither fulfill the criteria of similar group covariance matrices nor the criteria of containing a multivariate normal distribution as required by LDA. As we consider QDA and RDA, pure QDA is not feasible because of singularities in the covariance matrix.

#### RDA parameters

Descriptive RDA is performed over each training set for *λ*, shrinkage to common covariance, and *γ, shrinkage to diagonal of the covariance matrices, to estimate optimal parameters for an optimal CRR (correct recognition rate) as described by Lu et al. (2003)*[43]. We only test about ten combinations of *λ* and *γ* so this evaluation is rather crude. Best CRR under varying RDA parameters *λ* and *γ* is achieved with {*λ* = 0.99, γ = 0.001}. Larger numbers of descriptor variables (in relation to the observation count) would require a higher shrinkage parameter but with the selected rather small shape vector size of an average of 41 elements, there is no need to dispense with information by employing higher values for the shrinkage parameters.

#### Application of RDA under *k*-fold

Our application of RDA may be split into two steps. The first step involves deriving discriminant functions *df* based on the training set. These discriminant functions are used to describe group differences within a designated training set of objects. In a second step training set derived functions *df* are used to calculate discriminant scores *ds*for new objects given in the test set; as their group membership is not declared to the statistics software, that second step constitutes a test. The second step is used to establish a correct recognition rate (CRR) to quantify the classification power of the method.

#### CRR (correct recognition rate)

CRR is determined using six folds in a *k*-fold based evaluation of 6 test sets consisting of 18 objects respectively, previously neither used for variable selection nor for the optimal parameter search. Each time, we count the number of misclassified objects within the current test set. After RDA of all six partitions, we average resulting correct recognition rates.

### Robustness under added noise

Since injury peculiarities and operations during data acquisition and processing may deteriorate data accuracy, documenting decay of performance is of interest [44]. We add uniform noise within the range of *N* ∈[−*σ*,*σ*] to the original shape vector data. Each shape vector is changed according to a uniform noise generator. The resulting perturbed data matrix is analyzed again in the context of descriptive and predictive DA.

### Software and hardware

We use a 3D surface scanner (QTSculptor, Polygon Technology, Darmstadt, Germany) that provides vertex point resolutions below 100 *μm* with good depth-of-field and speed. Integrated software allows for segmentation, rendering, post-processing and exports to various 3*D* file formats. File export using the VRML 2.0 file format.

3D model manipulation and curvature are programmed in IDL (Interactive Descriptive Language, Exelis Visual Information Solutions, Boulder, Colorado, USA). 3*D* models are imported to IDL via a customized 3*D*data structure import parser.

For multivariate normal distribution testing, the package *R* is employed. For all other statistical evaluation and discriminant analysis, JMP (SAS Institute, Cary NC, USA) is used. Noise addition with Excel (Microsoft, Redmond, Washington, USA) and IDL.

## Results and discussion

### Descriptive RDA

We perform RDA with a data matrix containing 108 injuries. This database is partitioned 6 times into a training set of 90 and a test set of 18 injuries. Descriptive RDA is performed with a limited number of variables to estimate the best combination of parameters for RDA. The optimal parameter pair is *λ* = 0.99 and *γ* = 0.001 indicating a nearly linear DA with almost no shrinkage to the covariance matrix diagonal.

Descriptive RDA of all 6 training sets produce a perfect result [42]. Mahalanobis within-group distances are significantly smaller for each of the 108 modelled skin injuries than between-group distances. Receiver operating characteristic yields an ideal Area Under the Curve of 1.0 for all groups.

### Predictive RDA and CRR

For each of the 6 partitions in the *k*-fold validation, shape vector composition is optimized for the training set and then the CRR is calculated based on the number of misclassified items within the 18-element test set. We obtain 6 CRRs which subsequently are averaged yielding an overall CRR of 97.22%.

### Robustness under added noise

Adding uniform noise within the range of *N* ∈[−*σ*,*σ*] to both training and test sets prior to evaluation for all 6 partitions produces a degraded correct recognition rate of *CRR*_*N*_=71.3*%*.

### Alternative DA methods

Predictive LDA produces a CRR of 96.3% which is slightly less than the result of predictive RDA.

### Injury differentiation in the forensic context

Tools or weapons used to cause skin injuries may be distinguishable by their shape. While some items that are commonly employed to injure people are relatively deformable (e.g., fists, shoe soles, whips, cords used for ligature strangulation) others are relatively rigid (e.g., knives, axes, screw drivers, hammers, pliers). Even when instruments of similar appearance - two hammers, for example - are under investigation, it may be important to pay attention to details. Subtle defects of tool surfaces such as scratches or sharp edges are known to add specific features of injuries, such as tears, angles or abrasions [45]. Skin might exhibit variable amounts of deformation upon impact, depending on thickness, underlying tethering to bone, and depending on angle of impact.

Forensic juxtapositioning represents an illustrative method to investigate mostly rigid shape imprints. However, less rigid shape imprints are hard or impossible to match using that method. While forensic molecular biology dubbed “DNA fingerprinting” provides extremely helpful data to investigating authorities, genetic code comes pre-encoded. Statistical shape matching of skin injuries however can only be performed once analog injury shapes are digitally encoded. Digital shape encoding of injuries can offer the advantage of libraries [46] that can be queried automatically. One can exploit the encoding step to suppress noise while increasing descriptive uniqueness or discriminatory power. Statistical methods also can be applied in single instances for investigation of specific discriminatory questions.

This study bases its data on idealized, exaggerated and accurately digitized shapes. They are mostly easy to tell apart by eye. Yet our method shows no or little difficulties also in discriminating between smooth and textured strangulation marks. These could present a challenge were one to tell them apart by visual inspection.

We assume that injury shapes caused by distinct agents fall into distinct categories. While this assumption certainly is true for our plasticine block test set, it may not be seen as true for a range of real-life injuries. Abrasions, for example, could be graded just as burns can be graded. As that, some of their morphological aspects may be viewed as exhibiting continuity along some scale and for some concepts of causation and classification of injury, that could make it difficult to provide a clear distinction using methods such as discriminant analysis [41]. However, good forensic practice always provides - and has to provide - two or more hypotheses that are distinct, containing a complete set of explanations when combined and at the same time containing no overlap [47], as otherwise, not even a clear qualitative answer is possible. It thus is the requirement of a good legal investigation to provide correctly worded hypotheses as only for these, clear answers – e.g. by proper injury categorization – can be sought. Concerns regarding documentation quality and establishing quantified shape matches for forensic bite mark analysis have already been expressed [48].

Using the presented statistical method, it is possible to discriminate between group membership of injury-like surface deformations. Moreover, it is possible to obtain quantified information such as scores or likelihood ratios for a skin injury belonging to a particular suspected or assumed injury class given an alternative hypothesis. As a general rule in forensics, providing such quantified membership information is regarded as the job of the expert, while laying down cut-off levels for these likelihood ratios is seen as the task of the court or investigating legal body [49]. So far, only qualitative or evasive estimates were given by injury experts as to actual probability or likelihood ratios pertaining to shape matches.

However, we were recently confronted with the task of actually quantifying a morphological match for a district attorney’s office [50] where we applied similar shape vector based techniques for the identification of videoed finger shapes. There are a number of cases causative conclusions about injury shapes [51,52] seemed to provide determining angles for judicial verdicts.

### Morphological classifiers

With a best CRR of 97.22%, our classification results are comparable to what other investigators in the field of biometrics achieve. Results are obtained with injuries of different size, orientation in space, varied markedness and different placement of the injured region within each 3D mesh that underwent analysis.

We exploit curvature properties. More than a decade ago [53] and also for two-dimensional curves, curvature derived classifiers were found to exhibit robustness to scale, to rotation and to noise as a peculiar feature. Curvature derived information appears to successfully classify shapes also when extracted from two-dimensional images [13].

We use distance histograms for curvature range subsets of 3D surfaces. Histogram based techniques are known to exceed other approaches with respect to the power of biometric classification [15].

We exploit various scale levels of the 3D surface curvature while dealing with what appear to be partly fractal surfaces [17,54]: with limited resolution, we are not able to fully populate any scale level so we work with bins or range subsets to obtain at least minimally populated data. Using 3D curvature also allows us to exploit the whole injury data captured by 3D surface scanning; by restricting our analysis to contours (such as used for matching based on the Poisson equation [18,19]) we would not take advantage of shape details that are located at the center (and not the perimeter or contour) of the injuries that we examine; penetrating injuries, gravel rashes or lacerations all are not nearly as easily distinguished by their contours as they are by the geometry of their full surface (see Figure 2).

We optimize the statistical test. A relevant factor that contributes to the good result is that we optimize our choice of *λ* and *γ* parameters for RDA [43]. Better results may be obtained when analysing the covariance matrix structure according to [55].

We use excessive descriptive data to start with. Instead of starting with small shape vectors and extending them until a satisfying classification result was achieved, we start out with rather large curvature data based shape vectors containing 1284 descriptors. We then proceed to restrict the selection of variables down to an average of 41. For that, we use a signal-to-noise-type classifier that resembles a modified version of Wilks’ *λ* along with other data quality markers previously published by Richiardi et al. (2007) [37]. Employing an initially large array of shape descriptors to subsequently narrow features down to a small subset, also while maintaining a high correlate for SNR (signal-to-noise ratio) has become current practice also in forensics [56].

We use some particularly successful shape descriptors, as it turns out. The saddle-shape of hyperbolic points appears to constitute a relatively powerful descriptor given their rather frequent occurrence among the top classifiers in our study across all 6 partitions of the *k*-fold (Table 3, see also Table 1). Their intrinsic difference to other geometrical descriptors appears to be that only there do first order derivatives vanish at least in both two-dimensional projections [33] which is a feature that is exploited also for shape matching approaches based on Morse theory [57-59].

Another particularly successful concept seems to be the surface area and volume of the convex hull of vertices pertaining to particular curvature subsets (Table 3). Converting what appear to be abstract point sets to 3-dimensional surface structures (*γ*-surface [60], *α*-surfaces [61]) has long been recognized as important step in characterizing not just geometrical [62] but also data with no apparent geometrical significance [63]. Their usage for shape characterization does appear to introduce shape properties into statistical shape evaluation that were, so far, untapped which warrants further exploration [64].

## Conclusions

### Limitations of this study and application on real skin injuries

This study achieves a relatively good statistical discrimination of simulated surface injuries pertaining to six different categories. Each group was created to be highly distinct. In real life forensic pathology, textbook examples of real injuries are just as distinct as shown in Figure 1 and with that, our modeled plasticine models reflect clear cut reality well with regard to that aspect. However, real injuries also can appear to take a place between textbook categories and end up being hard or impossible to classify. In our view, that is a conceptual and not a technical problem. This study does not address this conceptual issue but focuses on data conditioning and feasibility evaluation.

First results of using similar descriptors based on 3D injury surface scans were promising [65-67]; descriptive RDA yielded very good results with almost perfect discrimination for clearly distinct injury categories when discriminating deep lacerations (caused by a hammer) from superficial abrasions (as consequence of contact with road tarmac). Data noise was not a relevant issue as we employed ample re-scan averaging and pre-scan treatment of the skin such as shaving and drying.

### Outlook

Our results are obtained with an optical 3D scanner calibrated to a close scanning range. It captures a lateral point resolution of about 100 *μm*. Noise massively degrades statistical discrimination so further improvement of data quality seems to be mandatory. The shape vector can be enhanced with additional automatically extracted quantifiable shape features, also with color information. First results obtained with two groups of 3D mesh objects captured from real skin injuries are promising [66].

Quantitative results to supplement expert considerations in forensic pathology might become more popular once they are reliable and once data analysis techniques become available as simple-to-use turnkey systems. This then might provide further incentive to document surface injuries three-dimensionally – a process that started with data acquisition through forensic photogrammetry [5-8], surface scanning [9,68] or CT-data-derived 3D surface extraction [69,70] to undergo subsequent analysis by virtual [71-74] or physical [75] forensic juxtapositioning spearheaded by the Virtopsy project [76-78] and recent anthropological developments [79] also using shape matching techniques [80-82].

## Competing interests

The authors declare that they have no competing interests.

## Authors’ contributions

ER conducted the practical work resulting in mesh model generation, refined shape vector elements, implemented statistical scripts and 3D mesh handling routines, drafted and revised the manuscript. MT authorized the study and gave final approval for the manuscript to be published. WS conceived the study, supervised scanner calibration and data acquisition, defined the Methods section, made substantial contributions to data analysis and interpretation and drafted and revised the manuscript. All authors read and approved the final manuscript.

## Pre-publication history

The pre-publication history for this paper can be accessed here:

http://www.biomedcentral.com/1471-2342/12/32/prepub
